# Labor Lost

**Published:** 1886-01

**Authors:** 


### LABOR LOST.

Some one sends to the editorial office of this journal an advertisement for insertion, without any address or name accompanying it. Of course, it cannot go in until we know from whom it is received Besides, business matters of this kind should always be forwarded to the business office in New York.

